# Nitrooxidative Stress and Neuroinflammation Caused by Air Pollutants Are Associated with the Biological Markers of Neurodegenerative Diseases

**DOI:** 10.3390/antiox13030326

**Published:** 2024-03-07

**Authors:** Abraham Alberto Ramírez-Mendoza, María Luisa Mendoza-Magaña, Mario Alberto Ramírez-Herrera, Zamira Helena Hernández-Nazara, José Alfredo Domínguez-Rosales

**Affiliations:** 1 Laboratorio de Biología de la Neurotransmisión, Departamento de Biología Celular y Molecular, Centro Universitario de Ciencias Biológicas y Agropecuarias, Universidad de Guadalajara, Zapopán ZC 45200, Jalisco, Mexico; abraham.ramirez1890@alumnos.udg.mx; 2 Laboratorio de Neurofisiología, Departamento de Fisiología, Centro Universitario de Ciencias de la Salud, Universidad de Guadalajara, Guadalajara ZC 44340, Jalisco, Mexico; luisa.mendoza@academicos.udg.mx; 3 Instituto de Investigación en Enfermedades Crónico Degenerativas, Departamento de Biología Molecular, Centro Universitario de Ciencias de la Salud, Universidad de Guadalajara, Guadalajara ZC 44340, Jalisco, Mexico; zamira.hernandez@academicos.udg.mx (Z.H.H.-N.); jose.drosales@academicos.udg.mx (J.A.D.-R.)

**Keywords:** particulate matter, air pollution, oxidative stress

## Abstract

Millions of people around the world are exposed to air pollutants, such as particulate matter 2.5 (PM_2.5_) and ozone (O_3_). Such exposure usually does not exclude these two types of pollutants and their harmful effects could be additive or synergistic. O_3_ is a highly oxidizing gas that reacts with the cellular environment just as PM_2.5_, triggering nitrooxidative damage. Once nitrooxidative stress overcomes the endogenous antioxidant system, an acute neuroinflammatory process is generated, and once it becomes chronic, it favors the formation of neurodegenerative disease markers. The presence of these markers becomes potentially dangerous in people who have a genetic predisposition and are at a higher risk of developing neurodegenerative diseases such as Alzheimer’s and Parkinson’s. Our experimental approach for nitrooxidative damage and neuroinflammation caused by air pollutants has focused on the exposure of rats to O_3_ in an isolated chamber. The hippocampus is the most studied brain structure because of its neuronal connectivity network with the olfactory epithelium, its weak antioxidant defense, and its fundamental roll in cognitive processes. However, other brain structures may exhibit a different degree of damage upon exposure to O_3_ and PM_2.5_, making their involvement an important factor in developing other CNS diseases. The age spectrum for augmented sensibility to air pollutants seems to mostly affect the pre-postnatal (autism spectrum) period and the elderly (neurodegenerative). Thus, a new approach could be the estimation of the damage caused by PM_2.5_ and O_3_ through a controlled exposure paradigm to determine the extent of damage caused by both pollutants.

## 1. Introduction

Human health depends greatly on the functionality of homeostatic protective mechanisms, which is at the first relay on maintaining the REDOX balance. This is related to the equilibrium between the presence, formation, function, and neutralization of reactive oxygen and nitrogen species (RONS), which in excess cause nitrooxidative stress, as well as their metabolites. These reactive species are counteracted by the activity of the endogenous antioxidant defense system (EADS), like catalase (CAT), superoxide dismutase (SOD), glutathione peroxidase (GPx), glutathione reductase (GR), and heme oxygenase-1 (HO-1) [1,2,3]. One of the sources responsible for the excessive RONS formation is exposure to environmental pollutants. Endogenously, the metabolic activity leads to the formation of RONS, and, usually, their rate of formation is neutralized by EADS [1], but a persistently high concentration of pollutants triggers RONS formation far beyond the neutralizing capacity of EADS. Recognized pollutants include pesticides, herbicides, drugs, heavy metals, toxic gases, particulate matter 10 (PM_10_), 2.5 (PM_2.5_), and ultrafine particulate matter (UFPM). Most of these pollutants share an anthropogenic origin and other organic pollutants like bacterial lipopolysaccharide (LPS) are frequently present [4,5,6,7,8]. Common air pollutants are nitrogen oxides (NOx), sulfur dioxides (SO_2_), and ammonia (NH_3_), among others, which are emitted in the gas phase; then, they undergo a photoreaction process (sun UV radiation) and, consequently, secondary pollutants like ozone (O_3_) are formed and their gaseous nature is preserved [9].

The air quality guideline levels (AQGL) established by the World Health Organization (WHO) in 2005 for PM_2.5_ in a 24 h exposure is 25 µg/m^3^ with an annual average of 10 µg/m^3^ as permissible, and for PM_10_, the daily exposure is 50 µg/m^3^ with annual average of 20 µg/m^3^. In 2021, these guidelines changed to 5 µg/m^3^ for PM_2.5_ in 24 h exposure with an annual average of 15 µg/m^3^, and 45 µg/m^3^ for PM_10_ in a daily exposure with an annual average of 15 µg/m^3^. Improving AQGL is a global challenge that has not been accomplished. With respect to O_3_, the AQGL in 2005 was 100 µg/m^3^ (50 ppb = 0.050 ppm) in a 24 h exposure and they remained unchanged in 2021 [10].

Particularly, our interest is focused on the harmful effects exerted by two air pollutants: PM_2.5_ and O_3_ in the central nervous system (CNS). These pollutants can induce RONS formation after their entry into the respiratory system and spread throughout the body, damaging the brain–blood barrier (BBB) and, finally, altering the brain homeostasis; all these events occur in an indirect route. These pollutants may also simultaneously reach the CNS through a direct route that initiates in the olfactory mucosa and reaches important brain structures like the olfactory bulb, entorhinal cortex, hippocampus, brain cortex, cerebellum, and brain stem [11,12,13,14].

To achieve the purpose of this review, we established a search and document analysis relative to the origin and nature of both pollutants PMs and O_3_, the nitrooxidative stress that each one induces, the transcriptional factors and signaling pathways involved, the alterations developed in the brain of human beings and animal models, the generation of neurodegenerative markers, and a final section that pretends to hallmark the importance of pharmacological resources to prevent and retard the damage process initiated by these pollutants.

## 2. Origen and Nature of PM

PM is classified depending on the particle size and the diverse substances contained in each PM. The main PM sources are oil refineries, factories, incinerators, forest fires, internal combustion motors, cement factories, and the construction industry, among others. Oil PM_10_ is mainly composed of particles ranging from 10 µm to 100 µm; however, the peak abundance is located between 20 to 30 µm. Despite this estimation, there are differences in their distribution among different geographical locations. Particles above 100 µm are not considered in this review because of their faster sedimentations on the ground surface due to the Earth’s gravitational force [15]. These PM_10_ are composed of fossil fuel combustion (black carbon or soot), volatile organic compounds (VOCs: benzene, aldehydes, carbon monoxide, ethylbenzene, and 1,3-butadiene, and others), mineral dust or ash (Mg, Al, Ca, Cr, Fe, Ni, Cu, Zn, and Pb, among others), sea or lagoon spray (H_2_O, ammonia, H_2_O_2_), inorganic aerosols (secondary), toxic gases, and organic matter (LPS) [16,17]. In highly populated cities, the most common source of fossil fuel combustion is the exhaust emission of internal combustion vehicles; thus, for a larger car density, a higher load of PM_10_ will be generated. The black carbon core (BCC) is less oxidized than the peripheral sheet by the effect of combustion. These particles do not penetrate the lungs, so their toxic effects are limited to the upper respiratory tract [17].

The composition of PM_2.5_ includes the (BCC), with adsorbed volatile organic compounds, material of organic nature, ions (sulfate, nitrate, ammonium), gases (NO_2_, CO, SO_2_) heavy metals, toxic gases, oxidized carbon, biological molecules, and VOCs, among others, which are particularly located in the corona zone of PM. PM_2.5_ possesses an aerodynamic size that enables their access to the pulmonary alveolar epithelium and causes local and systemic damage. These substances exhibit site- and time-dependent variations [17,18]. The black carbon particle may have a very diverse load of substances depending on local relative abundance; for instance, a heavier load with LPS may be expected in places where sanitary facilities are scarce and animal and human feces are on the ground surface. In other places, the proximity of factories with chimneys will contribute to the load of toxic gases, ammonia, or other harmful substances.

The UFPM (<0.1 µm) exhibit a smaller diameter than PM_2.5_ and they also easily reach the alveolar epithelium and may cause damage [19]. In spite of its small mass, UFPM may account for higher particle counts than PM_2.5_ and PM_10_. Thus, in terms of health hazards, UFPM can be considered among the most reactive because their surface allows great adsorption. UFPM easily reaches the alveolar epithelium and penetrates this barrier reaching blood circulation, throughout which they can systemically spread to all tissues and organs. UFPM may contain important amounts of toxic gases like NOx, O_3_, SO_2_, NH_2_, trace metals, and organic matter (e.g., LPS) [19].

## 3. Nitrooxidative Stress by PM_2.5_ in the CNS

Controlled exposure to PM_2.5_ in experimental models is necessary to identify and characterize the initial changes that lead to systemic injury or complications and brain damage. In this context, the effect of PM_2.5_ has been approached in a model of single- and three-time repeated exposure model by intranasal instillation. Interestingly, NO was the initial oxidant molecule generated as inducible nitric oxide synthase, which was overexpressed in lung lavages 30 min after a single exposure, causing vascular endothelial dysfunction [20]. This event was followed by a second round of oxidative stress evidenced by the formation of malondialdehyde (MDA) as an indicator of lipoperoxidation caused by other oxidant species like H_2_O_2_. Thus, PM_2.5_ exposure induces nitrosative stress (NS) caused by nitrogen reactive species (RNS), earlier than the oxidative stress (OS) caused by oxygen reactive species; they both continue the production of nitrooxidative stress (NOS) caused by reactive nitrogen and oxygen species. These observations suggest that PM_2.5_ induces nitrooxidative stress in a sequenced manner [20]. Thereafter, Piao et al. [21] reported that exposures to PM_2.5_ by intranasal instillation induced oxidative stress and inflammation in a mouse model of allergic rhinitis through the activation of the Nrf2/NFⱪB signaling pathway. However, a major concern arises considering PM_2.5_ caused spatial learning and memory impairment, affecting inquiring ability, and sensory function. These alterations were supported by ultrastructural analysis where mitochondrial changes, myelin sheet disarrangement, and neuronal apoptosis occurred [21,22]. The exposure of rats to an experimental load of ambient dusty PM from 200 to 500, 500 to 2000, and 2000 to 8000 µg/m^3^ caused BBB damage, OS, increased concentration of inflammatory cytokines, and brain edema. These changes were associated with impaired spatial memory and hippocampal long-term potentiation (LTP) [23]. It has been proposed that PM_2.5_ is capable of inducing changes in platelet parameters, megakaryocyte activation, OS, and neuroinflammation that lead to the development of ischemic stroke, thus becoming an additional risk factor aside from those previously described [24]. PM_2.5_ exposure has also been associated with an increase in cases of children affected by autism spectrum disorder [25].

## 4. Transcriptional Factors Activated by PM

The exposure to PM_2.5_ induces the formation of RONS which are responsible for a milliard of consequences that alter homeostasis. Some of these consequences include the activation of transcriptional factors like Keap1-Nrf2-ARE, NFκB, TLR, and MAPKs, which modify the expression of genes involved in important mechanisms to recover homeostasis through adaptive changes [26].

The axis Keap1-Nrf2-ARE is an important signaling pathway that, when activated, induces the expression of antioxidant and cytoprotective gene responses. The oxidation of serine residues in Keap1 by RONS allows the release of Nrf2 from Keap1 and Cul3, which are degraded by the proteasome. Once Nrf2 is released, it undergoes nuclear translocation, forming a heterodimer with Maf and binds to the antioxidant response elements (ARE), and thus the expression of antioxidant enzymes and cytoprotective proteins increases. If Nrf2 is not released Cul3-Keap1 regulates Nrf2 polyubiquitination leading to its proteasomal degradation [27]. The antioxidant response mediated by Nrf2 activation includes the expression molecules that are part of the AEDS like CAT, SOD, and GR. However, this can be overcome if the exposure to exogenous oxidant agents, like PM or O_3_, is intense and prolonged [28].

The other transcriptional factor that plays an important role in the inflammatory response is NFκB. In healthy cells, this factor is localized in the cytosol as a heterotrimeric complex formed by the subunits p65/p50 with its bound inhibitor subunit (IκB), which avoids NFκB nuclear translocation. However, when an adequate signal occurs (e.g., oxidative damage), IκB is rapidly ubiquitinated and degraded in the proteasome; once released, the p65/p50 dimer undergoes nuclear translocation and binds to its response elements, inducing the expression of diverse molecules including inflammatory cytokines and pro-oxidative enzymes among others [29]. A remarkable protein induced by NFκB activation is KEAP1, which plays an important role in regulating the activation of the transcriptional factor Nrf2 [30]. During the exposure to PM, NFκB becomes activated by OS, which induces the activation of its kinases, IKKα and IKKβ, while IKKγ (NEMO) acts as a regulatory subunit [31]. These kinases are also activated by the TNF receptor, Toll-like receptor, and interleukin receptors in the canonical pathway, while the non-canonical activation requires the stimulation of specific TNF receptors that lead to the recruitment of TRAF2 and TRAF3 [32]. This pathway continues with the activation of NFκB-inducing kinase. RONS are also capable of activating NFκB through alternative phosphorylation of IκBα (NFκB inhibitor). The phosphorylation by RONS is mediated by casein kinase II, particularly in tyrosine residue 42 and other tyrosine residues in IκBα [32]. Furthermore, RONS like H_2_O_2_ can activate IKKs through the formation of disulfide bonds between cysteine residues 54 and 347. Thus, the release of NFκB dimers (p50/p65) translocates to the nucleus and binds DNA, increasing the expression of inflammatory cytokines (TNFα) and interleukins (IL-1β, IL-6, IL-11, IL-17), as well as pro-oxidant enzymes (iNOS, COX-2, LOX-5, LOX-12) [32]. NFκB activation by RONS induces the expression of inflammatory cytokines, which, after binding to their receptors, may overactivate NFκB, leading to an amplified pro-oxidant and inflammatory response.

An important feature of the crosstalk between Nrf2 and NFκB is that, upon activation of NFκB, the expression of Keap1 is increased, leading to its binding to Nrf2 and its consequent proteasomal degradation and the decreased expression of antioxidant enzymes [30].

## 5. Human Brain Damage by PM

Studies on the effects of highly polluted air in megacities have been reported by Calderón-Garcidueñas since 1992 [33], where total pollutant load was associated with a variety of alterations in diverse human and dog tissues and organs [34]. However, it was not until 2015 that PM_2.5_ was associated with specific disease markers for obesity, Alzheimer’s disease (AD), non-Alzheimer’s dementia (N-AD), Parkinson’s disease (PD), amyotrophic lateral sclerosis (ALS), and others [4,35,36]. Furthermore, PM_10_, SO_2_, NO_2_, and NO have been associated with worsening multiple sclerosis (MS) outcomes, hypothesizing that oxidative stress and inflammation damage BBB lead to chronic neuroinflammation. These events could be followed by an immune attack reinforced by transcriptional factors involving activated microglia (microgliosis) that attack neuronal tissue and contribute to an important decrease in self-tolerance and possible production of autoantibodies [37,38]. PM air pollution has also been associated with pre and postnatal CNS damage, particularly the increasing incidence of autism and autism spectrum disorder (ASD) that has been reported [25,39,40].

Prenatal exposure to PM in highly polluted urban zones has been associated with developmental retardation of brain maturation processes [41]. Such retardation may contribute to the onset of neurological conditions like schizophrenia and ASD that are clinically diagnosed in early childhood. Furthermore, the alteration in brain development is evidenced by decreased intellectual performance, behavioral alterations, cognitive disorders, memory consolidation, and motor language difficulty [42,43], and boys are apparently more susceptible than girls.

When exposure to PM_2.5_ increases between 5 months before birth and 1 month after birth (perinatal), additional changes occur and neurodegenerative disease markers are detected [44,45].

The maturation of the human brain in boys apparently requires an extended period; thus, the EADS is not quite efficient, and exposure to PM_2.5_ and NO_2_ causes profound damage, as previously documented. The detrimental effects were significant in memory and verbal performance. Furthermore, affectation in global cognition, including numeric and motor skills were affected mainly by NO_2_ [43]. The most important effects could be due to oxidative stress, systemic and neuro-inflammation, as well as decreased fetal growth. These differential adverse effects may be due to a wider sensitive window for boys and narrower for girls; could it be attributable to a faster maturity process of the EADS for the female brain? In experimental models, similar observations have been documented with the additional control of sex and exposure period (pre and/or postnatal). Learning, memory, and behavioral flexibility were affected leading to an impulsivity-like behavior. There were differences in the amino acid pool probably related to sexually differentiated neurotoxicity, in spite of microglia being persistently activated in both males and females [19,46].

Exposure to PM_2.5_ in early postnatal life leads to an increased risk of developing attention deficit hyperactivity disorder (ADHD) and ASD. As exposure to PM_2.5_ in early postnatal life occurs in a continuous manner, the alterations in neuronal development are difficult to discriminate from other potential factors [47]. Furthermore, the standardization of experimental models faces serious difficulties due to the neurodevelopmental velocity in animal models (28 days) versus the three years required in human beings. Thus, even the sequence of damage to involved molecules and the response capacity of the organisms may be different in both cases.

The effects of air polluted with PM in late childhood (3 years and over) have been associated with the onset of anxiety and depression symptoms, and ADHD also increases. Thus, it would be important to develop strategies to mitigate the impact of air pollution on children’s health. As exposure of children becomes harder to control, the risk of exhibiting improper behavior and emotional distress increases. When exposure time increases in childhood, the incidence of criminality may also increase for teenagers. These and other issues regarding the consequences of uncontrolled inhaling of PM using experimental models or data obtained and analyzed from human populations require further study [47,48].

As people arrive at adult age, the exposure level to air pollution increases due to economically remunerated work activities, which require the use of private or collective transportation. Furthermore, the acquisition of addictions like smoking, the use of domestic PM generators (stoves and ovens), and the establishment of unhealthy lifestyles, increase the risk of developing chronic degenerative diseases [15]. Such pathological conditions can cause disability and premature retirement. Moreover, these diseases share common features in the deepest changes at the molecular level like oxidative stress and chronic inflammation. This also applies to neurodegenerative diseases, which, at a late stage, lead to dementia, loss of personality, high economic expenses (caregivers and hospital), social conflicts, and patients becoming a heavy burden to relatives [49,50,51,52].

## 6. Neurodegenerative Markers Induced by PM

As previously mentioned, both PM_2.5_ and UFPM can deeply diffuse through the respiratory tract and both possess a BCC; thus, the adsorbed molecules may vary depending on their presence and relative abundance in the inhalable air. These PMs find the first entry portal to the CNS in the olfactory neurons residing in the olfactory mucosa. In humans, the incorporation of UFPM seems to be lower than 3.5%; however, in a mouse model exposed to synthetic BCC, its intracellular presence in the olfactory bulb neurons has been associated with an increased release of glutamate and glycine, together with inflammatory cytokines, after 6 and 11 h of exposure [53]. This excitotoxic and inflammatory scenario could occur in human populations as endothelial hyperplasia, accumulation of PM, and increased Aβ and α-synuclein immunoreactivity in neurons and glial cells, as it has been reported in the olfactory bulb neurons from young citizens of the Mexico Metropolitan City (MMC) [54].

Simultaneously, the systemic route contributes to the diffusion of UFPM and after interacting with alveolar macrophages they initiate a process of OS and inflammation. The UFPM, RONS, and inflammatory cytokines reach the brain through the blood circulation and disrupt the integrity of the BBB, damage endothelial cells, and spread oxidative damage, neuroinflammation, Aβ deposition, and neurofibrillary tangles [55].

Exposure to high concentrations of PM_2.5_ has been associated with neuronal Aβ plaques in patients with dementia or mild cognitive impairment. Furthermore, in young adults and children, the Aβ plaque deposition precedes a neuroinflammatory status. Beta-site amyloid precursor protein cleaving enzyme (BACE1) is overexpressed by exposure to PM_2.5_. It acts on the amyloid precursor protein and generates Aβ. BACE1 can be inhibited by miR-574-5p; however, NFκB activation downregulates its expression. Thus, when nitrooxidative stress caused by PM_2.5_ activates NFκB and BACE1 generates Aβ. Consequently, miR-574-5p exhibits protective effects on sinaptogenesis, and improves cognition, learning, and memory after exposure to PM_2.5_ [56].

Hippocampal and glutamatergic neurons are highly susceptible to nitrooxidative stress caused by PM. Considering glutamatergic neurons are more prone to exhibit excitotoxicity upon PM exposure, their viability decreases in a dose-dependent manner with activation of caspase 3 [57].

Dopaminergic neurons are also highly susceptible to nitrooxidative stress; as a consequence, PM induces neuronal loss through RONS formation and is reinforced with the generation of O_2_^−^ by microglial cells. Thus, PM_2.5_ has been associated with an increased incidence of PD and is considered an important risk factor [58].

Nitrooxidative stress and neuroinflammation are central generators that create a vicious cycle or damage spiral. The adsorbed oxidant pollutants that surround the BCC and the BCC by itself are potent inducers of RONS, which activate NFκB leading to the expression of inflammatory interleukins and cytokines [59,60].

The intracellular changes induced by PM include mitochondria abnormal morphology, dissociation of mitochondria, and endoplasmic reticulum contacts with an accumulation of intracellular PM. Mitochondria changes are pore opening with permeability transition, reduction of mitochondrial potential, decreased ATP production, and low mtDNA copy number [61].

Table 1 summarizes the effects of PMs in animal models and human populations which were analyzed in the sections above.

## 7. Origen and Formation of O_3_

O_3_ is a triatomic molecule formed by oxygen, and this allotrope located at the stratosphere level confers anti-UV protection. Contrarily, O_3_ at the tropospheric level (ground level) causes severe damage to living beings, including humans. High-income countries exhibit the highest O_3_ rate formation; however, developing economies share high O_3_ exposure levels, too. Tropospheric O_3_ also affects climate as it alters plant growth and survival. The formation of O_3_ implies the chemical interactions of nitric oxides (NO_*x*_) with NO_2_, VOCs, O_2_, and CO. The presence of these gases in the troposphere with a high incidence of solar UV radiation (late spring/early summer) leads to an intense and persistent O_3_ formation [62]. However, countries located between the 30th parallel receive more solar radiation at a perpendicular angle, which leads to sustained O_3_ formation throughout the whole year. Daily O_3_ formation is significantly increased between 10:00 to 17:00 h. This period varies depending on the geographic latitude [62]. The most important feature of O_3_ is its capability to induce the formation of RONS when interacting with a living tissue microenvironment.

## 8. Nitrooxidative Stress by O_3_

The O_3_ exposure induces a broad spectrum of local, systemic, and CNS alterations. The interaction of O_3_ with organic substrates containing double bonds leads to the formation of ozonides, which further leads to the formation of hydroperoxides. Thus, as O_3_ is not a radical species, it functions by inducing the formation of RONS [63]. In this context, direct and indirect processes occur during the formation of RONS by O_3_. A direct process is due to the oxidation of biomolecules generating radical species that initiate a chain reaction and is linked to the indirect process. The indirect process implies the generation of nonradical cytotoxic metabolites generated by RONS, which cause deleterious effects. This phenomenon arises from a toxicity mechanism in which O_3_ engenders an instigating production of RONS that possess the inherent capacity to inflict cellular damage by initiating membrane lipoperoxidation, and carbonylation/nitrosylation of proteins [3,64]. The oxidative byproducts resulting from phospholipid-mediated lipid peroxidation like F2-isoprostane (F2-isoPs) and F4-neuroprostane, derived from arachidonic acid, should be highlighted due to their increased concentration in brain regions with amyloid β plaques, neurofibrillary tangle formation, and abundant neuronal loss [65,66]. Moreover, it has been elucidated that these oxidative processes give rise to harmful aldehydes, namely, 4-hydroxynonenal (4HNE) and MDA, which exert deleterious effects on both the structure of the plasma membrane and mitochondrial integrity [67]. Consequently, these oxidative events affect cellular integrity and homeostasis, thus compromising mitochondrial functionality.

Proteins are also damaged by RONS, and carbonylation as well as nitrosylation occur, as previously reported [11,64]. A plethora of physiological and pathological processes have documented the role of protein nitrooxidative damage, which led to the generation of protein misfolding and impairment of the proteasome function (proteostasis). These events have been deeply associated with aging/senescence as a natural or experimentally induced process [68,69,70,71]. Also, nitrooxidative damage has been documented as caused by cigarette smoke and PM [72], as well as by O_3_ leading to the formation of 3-nitrotyrosine (3-NT) [64]. Nitrooxidative stress contributes to altered mitochondrial biogenesis (fission) and causes bioenergetic failure, particularly when NO is formed at excessive levels during Aβ oligomerization or when glutamate receptor (NMDA subtype) is overactivated; these events are involved in AD physiopathology [73]. Furthermore, the excessive production of NO, together with the superoxide anion (O_2_^−^) generated during normal mitochondrial function, leads to the formation of peroxynitrite (ONOO^−^) [73].

In the case of AD, biomarkers identified, validated, and related to nitrooxidative stress include F2-IsoPs, carbonylated proteins, MDA, and 3-NT in conjunction with increased expression of heme-oxygenase 1 (HO-1); however, its activity is affected by other molecules like Aβ. This scenario resembles the induction by O_3_ and PM_2.5_, whether it occurs independently or simultaneously [74].

The accumulation of misfolded protein aggregates is another important feature in neurodegenerative diseases like AD and PD. This process is also found as a physiological event related to the aging process; however, when it occurs in early life, it is considered a proteinopathy [75]. Proteins fold into a native conformation as they achieve a correct and more stable array to exert their function. If the folding process occurs in an altered manner, proteins accumulate in the cytoplasm and should undergo degradation by the proteasome; if this degradation system has been damaged by a nitrooxidative stress, then a condition termed proteostasis is established and misfolded proteins accumulate without elimination [75]. The effect of O_3_ in different exposure approaches has been reported and the folding pattern resembles the protein folding occurring in neurodegenerative diseases. The accumulation of misfolded proteins has been detected at the intracellular and extracellular levels. Misfolded pattern formation of Aβ_1–42_ caused by repeated low exposure to O_3_ includes a conformational change from α-helix to a mixture of α-helix with a β-turn in an unordered fashion [76,77]. This effect caused by O_3_ was confirmed by Mendoza-Magana et al. (2021) in a short-term neurotoxicity model caused by O_3_ at a concentration of 0.7 ppm for 1, 2, 4, and 8 h. Neurodegeneration was evaluated through histochemistry using modified de Olmos silver and Fluoro-Jade staining. Both stains evidence the accumulation of cytoplasmic misfolded proteins, which was detected after 2 h post-exposure reaching a maximum detection after exposure for 8 h [64].

The pathophysiological alterations observed in the CNS are attributable to its heightened susceptibility to oxidative stress incited by O_3_ encompassing neurochemical perturbations, cognitive deterioration, diminished motor functions, cephalgia, functional impairment, and neuronal degeneration [78].

## 9. Transcriptional Factors Activated by O_3_

The most studied transcriptional factors upon exposure to O_3_ are Nrf2, NFκB, MAPK, and NLRP3. The OS caused by O_3_ triggers the activation of these important signaling pathways, and the consequences of their activation are closely related to the development of neurodegenerative diseases [63]. There is clear evidence that O_3_ induces activation of NFκB through the activation of IKK by RONS [11,79,80]. The consequence of NFκB activation has been mentioned before and long-term exposure to O_3_ leads to the establishment of a chronic inflammation process associated with the generation of biological markers of neurodegeneration [80].

The Nrf2 transcriptional factor is activated to reinforce the antioxidant endogenous system against an OS, by increasing the expression of antioxidant enzymes and molecules involved in cell repair. However, if the oxidative damage is severe and maintained long-term, the Nrf2 becomes inactivated by overexpression of Keap1 induced by NFκB activation [30]. As Keap1 complexes with Cul3 that binds to Nrf2, the proteasome elimination of Nrf2 is increased, and its intranuclear presence decays; consequently, the expression of antioxidant enzymes decreases.

By far, it becomes important to revert both ozone effects on these transcriptional factors attempting to restore redox balance. Thus, the search for pharma/nutraceutical resources to achieve this goal is critical.

## 10. Brain Damage by O_3_

It should be noted that the generation of neurodegenerative markers has been detected in neuronal tissue obtained from experimental models, sentinel animals (feral dogs), and deceased people. The data from animal models are more robust than those from sentinel animals or humans, but these are also important. In experimental models, generating an artificial atmosphere with an established and constant O_3_ concentration (dose) is a relatively easy process. The design and construction of exposure chambers follow a clearly understood concept. The chambers are made of acrylic hermetically sealed to allow the continuous monitoring of the mixed flux of O_3_ with O_3_-free air to secure a given dose of O_3_. The O_3_ expelled from the chamber after exposure should be neutralized with a filter before it is released into the outer air. Animals are subjected to a habituation period (5 to 8 days) to avoid stress caused by their allocation and handling in the exposure chamber. The aim of this procedure is to prevent oxidative stress and alterations during manipulations of the experimental groups [81].

Even though O_3_ has been considered a therapeutic approach by inducing the reinforcement of the antioxidant response, blaming it as a causative agent, when inhaled, for AD and other neurodegenerative diseases, its use has been controversial, as the hallmarks classically recognized for each one are controversial as well. However, two indisputable events are constantly developed during the beginning and ongoing of such conditions: oxidative stress and chronic inflammation. It is well known and documented that O_3_ exhibits a strong oxidative capability and its interaction with living beings infringes an alteration to the redox balance through the formation of RONS [78,82]. An excessive short or long-term RONS formation easily overcomes the EADS and exhausts the capability of Nrf2, the transcription factor responsible for inducing the expression of antioxidant enzymes. Additionally, Nrf2 induces the expression of BACH1, which decreases the binding of Nrf2 to the ARE response elements [83,84,85].

It is necessary to consider that inhaled O_3_ at higher concentrations than those established as safe by the WHO and local government regulations, can cause oxidative damage (0.05 vs. 0.5 ppm). With this in mind, the following analysis could discuss the capacity of O_3_ to induce the formation of Aβ and its impact on cognitive performance. As reported by Hernández-Zimbrón et al. [86], the exposure of Wistar rats to an O_3_ concentration of 0.25 ppm for 4 h daily during 15, 30, 60, and 90 days provoked a significant accumulation of Aβ42 at 60 and 90 days in the hippocampus, as demonstrated by immunohistochemistry and Western blot; this occurred with a concomitant decrease in Aβ40 accumulation. Simultaneously, they found mitochondrial accumulation of Aβ42, as it co-localized with COX1 in the dentate gyrus. Previously, they demonstrated that exposure to O_3_ increased lipid peroxidation and accumulation of superoxide anion, which is related to energy failure in AD [86]. More recently, they reported the formation of Aβ42 in the hippocampus of Wistar rats exposed to O_3_ and analyzed the global conformation changes by Raman spectroscopy. They found that O_3_ caused a decrease in the α-helix of the secondary structure and an increase in the β-sheet conformation. Thus, the OS caused by O_3_ induced changes in the folding process of Aβ, which would finally acquire a final folded structure as β-sheet, resembling the form found in AD immunohistochemistry of deceased patients [76]. The increased immunoreactivity for Aβ42 occurs in a time-dependent mode and that predominance of the α-helix structure decreases along the exposure time and is replaced by an unordered β-helix structure embedded in an environment where OS occurs along with increased expression of COX1, diminished activation of Nrf2, and augmented activation of NFκB. This scenario resembles the physiopathology of AD [76]. The neurodegenerative changes including the increased concentration of oxidized and nitrosylated biomolecules (membrane phospholipids, carbohydrates, proteins, DNA, RNA), mitochondrial dysfunction, misfolded protein accumulation, endoplasmic reticulum stress, apoptosis, and so on, coincides with a cellular response to a harmful condition that leads to neuronal death [63,77,87]. These changes are behaviorally reflected as memory loss, cognitive decline, locomotor impairment, as well as neuropsychiatric symptoms like apathy, anxiety, and depression [88,89]. However, there are reports in which the exposure to ozone of double transgenic mice (APP/PS1) [0.8 ppm/7 h/day/5 days, followed by 9 days for 8 cycles of recovery period]. There was no increase in Aβ42 load in the transgenic male mice exposed to O_3_ versus O_3_ unexposed transgenic male mice. The same phenomena occurred when transgenic female mice exposed to O_3_ were compared to transgenic female mice not exposed to ozone. However, female transgenic mice exhibited higher Aβ42 deposition than male transgenic mice, and O_3_ exposure did increase its deposition [87]. Interestingly, the detection of 4HNE protein adducts was higher in male transgenic mice when compared to female transgenic mice. Similarly, apoptosis in male transgenic mice exposed to ozone was increased when compared with female transgenic mice exposed to O_3_. This was attributable to a lower level of antioxidant defense in male transgenic mice versus female mice when both were exposed to O_3_. Moreover, memory/learning function declined in male transgenic mice exposed to O_3_, but not in female mice. Thus, the authors propose that O_3_, by itself does not cause AD and, in their model, male mice are more susceptible to oxidative stress and apoptosis than female mice [87]. In this scenario, the high Aβ deposition in the hippocampi of female mice does not directly correlate with the abundance of 4HNE-protein adducts, induction of antioxidant response, and memory function. An important difference in the experimental exposure design that should be addressed is that the experimental exposure to O_3_ includes a recovery period of 9 days in an 8-cycle mode. This could exert an important influence to permit a natural clearance of an Aβ load induced immediately after damage caused by O_3_; however, it is probable that chronic exposure to O_3_ (60–90 days) may induce deeper harmful changes and could induce the establishment of an AD-like physiopathology caused by O_3_ exposure [76,77,86,90].

Other alterations caused by O_3_ at a low dose (0.25 ppm/4 h daily for 60 and 90 days) have been reported, like endoplasmic reticulum stress being linked to the induction of apoptosis through activation of caspase 12 [77]. These observations reinforce the role of O_3_ as an inducer of tissue damage similar to that observed in neurodegenerative diseases like AD [77]. In contrast, with this same O_3_ exposure scheme, the dendritic spine density was decreased at 15, 30, 60, and 90 days of exposure, suggesting that oxidative stress induces deafferentation in the hippocampal CA1 region affecting the integration of multimodal information from the entorhinal cortex. The thin spine ratio decreased at 15 and 90 days of exposure; meanwhile, the mushroom spine ratio exhibited a dramatic decrease at 90 days versus 15 days of exposure. At 90 days of exposure to ozone, the branched spine ratio decreased [90]. The multi-head spine ratio decreased only at 15 days of exposure to O_3_. In this context, the learning and memory processes are affected and cognitive deficit reflects a physiological deterioration of the brain [90]. The exposure to O_3_ was at a concentration of 0.25 ppm for 4 h daily during 7, 15, 30, 60 and 90 days. The damage was evaluated in substantia nigra that exhibited an increased concentration of carbonylated proteins in a time-dependent manner. The release of cytochrome C from mitochondria occurred from day 15 to 90 reaching a maximum level on day 30. Similarly, the astrocytosis increased from day 7 to 90 with a maximum activation on day 30. The activation level for microglial cells was assessed with an antibody against the ionized calcium-binding adaptor molecule 1 (Iba1), which occurred on day 60 [79]. NFκB was strongly detected in the nucleus on day 30 by immunohistochemistry, while the maximum level of COX2 was detected on day 15 [79]. The inflammatory response was dysregulated with extensive oxidative stress, dopamine oxidation, and neuronal cell death, as it commonly occurs in Parkinson’s disease [79,91,92].

Besides the evidence of molecular biomarkers for AD-like neurodegeneration, which can be estimated through analytical techniques, these may exhibit considerable variability and their impact on disease outcome could be undetermined. Cognitive decline is the most important feature in the development of AD-like dementia in the elderly population due to the demand for caregivers, nursing, and medical assistance which are more demanded [49]. A longitudinal study that analyzed the impact of O_3_ and PM_2.5_ exposures on cognitive decline was performed by Cleary et al. [93]. They found that cognitive decline increases with increasing concentrations of O_3_, and, annually, the Mini-mental Status Examination declined by 1.4, 1.3, and 1.1, and the Cognitive Dementia Rating Sum of Boxes also declined by 1.1, 1.0, and 0.9; these declining rates occurred in high, medium, and low O_3_ concentrations, respectively. But, no cognitive differences related to the exposure to PM_2.5_ were observed. Exploring the association of cognitive decline rate with the presence of an APOE4 allele in the people exposed to O_3_ and PM_2.5_, the rate was faster in those carrying at least one E4 allele, compared to those not harboring such allele. The impact of PM_2.5_ was not significant in people without the E4 allele [93,94].

Systemic chronic degenerative diseases have been proposed as important factors that worsen the progression of AD. Since diabetes mellitus type 2 (DMT2) has been diagnosed in over 80% of patients with AD and the physiopathology of both pathological entities usually overlap, increasing suspicion of an intimate relationship is growing. They share common features like insulin resistance, amyloidogenesis, oxidative stress, inflammation, and neuronal apoptosis, among others [95]. The disrupted function of many signaling pathways in AD and DMT2 are overlapped in the establishment of both diseases. Thus, there is important evidence of the intimate link between the physiopathology of AD and DMT2, as the risk of developing both may be increased by inhaled ozone in highly polluted cities. This risk decreases in rural environments where ozone levels are under harmful concentrations [96].

O_3_ exposure has been pointed out as an important risk inducer for AD (recently considered as diabetes type 3) and DMT2. Diverse and innovative proposals have considered the noxious effects of O_3_ on glucose metabolism directly causing insulin resistance, hyperglycemia, hyperlipidemia, oxidative stress, chronic inflammation, and endoplasmic reticulum stress [97]. These changes are associated with an increased phosphorylation of insulin receptors (IRs) and decreased activity of the insulin-degrading enzyme (IDE) [95]. Besides regulating insulin levels, IDE also decreases the formation of Aβ contributing to its clearance. Consequently, when O_3_ alters glucose metabolism the accumulation of Aβ increases. At the intracellular level, Aβ alters mitochondrial function and decreases ATP synthesis; meanwhile, at the extracellular level, it polymerizes forming Aβ oligomers and fibrils that accumulate in the outer side of neuronal membranes binding to IRs competing with insulin and decreasing glucose internalization [86]. The extracellular accumulation of glucose may lead to increased levels of advanced glycation end products (AGEs) that after binding to their receptor will initiate the activation of intracellular signaling pathways including activation of NFκB contributing to the establishment of a chronic inflammatory state [95,98,99].

Studies with experimental models report that the exposure to O_3_ at 0.8 ppm concentration for 16 h in conventional adult Wistar rats caused an increase in fasting glucose and insulin levels. HOMA-IR also increased after O_3_ exposure, reflecting an impairment in insulin sensitivity. Furthermore, the insulin released by glucose stimuli was not altered by inhaled O_3_. This suggests that glucose intolerance caused by O_3_ was not due to a decrease in insulin release, but to peripheral IR [97]. The signaling pathway affected was protein kinase B/Akt phosphorylation in muscular cells where IR occurs, but it was not affected in hepatocytes nor in white adipocytes. The alveolar fluid did not exhibit increased levels of inflammatory cytokines, but oxidative stress markers HNE, MDA, HHE, and protein carbonyl levels were increased [97]. Protein carbonyls were also detected in muscular cells. Thus, oxidative stress seems to be a major contributor to IR contributor induced by O_3_ and the accumulation of lipid peroxidation metabolites can induce ER stress and the activation of the JNK pathway. If O_3_ exposure is repeatedly applied and lung inflammation, oxidative stress, and IR are perpetuated, then this condition, in conjunction with an inadequate diet, sedentary lifestyle, and practicing outdoor physical activity with the presence of PM, could, importantly, contribute to the development of DMT2 [97].

## 11. Neurodegenerative Markers Induced by O_3_

The exposure to tropospheric O_3_ at toxic concentrations leads to a spectrum of important alterations. These alterations depend on the exposure dose, duration, frequency, toxicity, and susceptibility of each organism. Nonetheless, they may also increase if simultaneously there is exposure to other pollutants, such as industrial chemical waste. Consequently, neurodegenerative markers may vary depending on the life stage, such as prenatal, perinatal, childhood, adulthood, and old age [81]. However, in a general landscape, the following alterations could be detected: retardation or alteration of neuronal structure maturation at the prenatal age; changes in neurotransmission involving synthesis, transport, and release of neurotransmitters, including their binding to receptors; oxidative damage which affects membrane phospholipids, carbonylation and nitrosylation of proteins, which affects their structure, conformation, and function; formation of protein and nucleic acid adducts; accumulation of misfolded proteins, mitochondrial damage that leads to energy failure; and DNA and RNA oxidation affecting transcription and translation efficiency and inducing nucleotide substitution [68,81,86,91,100]. The brain tissue generates a response in an attempt to restore normal function. This response includes the activation of astrocytes and microglial cells. The astrocytosis implies an increased immunoreactivity to GFAP that is accompanied by an increased secretion of NGF, VEGF, BDNF (the significance of this response deserves investigation), IL-1, IL-6, and TNF-α, with decreased secretion of IL-10. Microglial activation implies the acquisition of phagocytic activity with increased production of RONS and secretion of inflammatory cytokines. These changes are meant to be beneficial to the neuronal tissue; however, under chronic activation, they become harmful [63,101,102].

**Figure 1:**
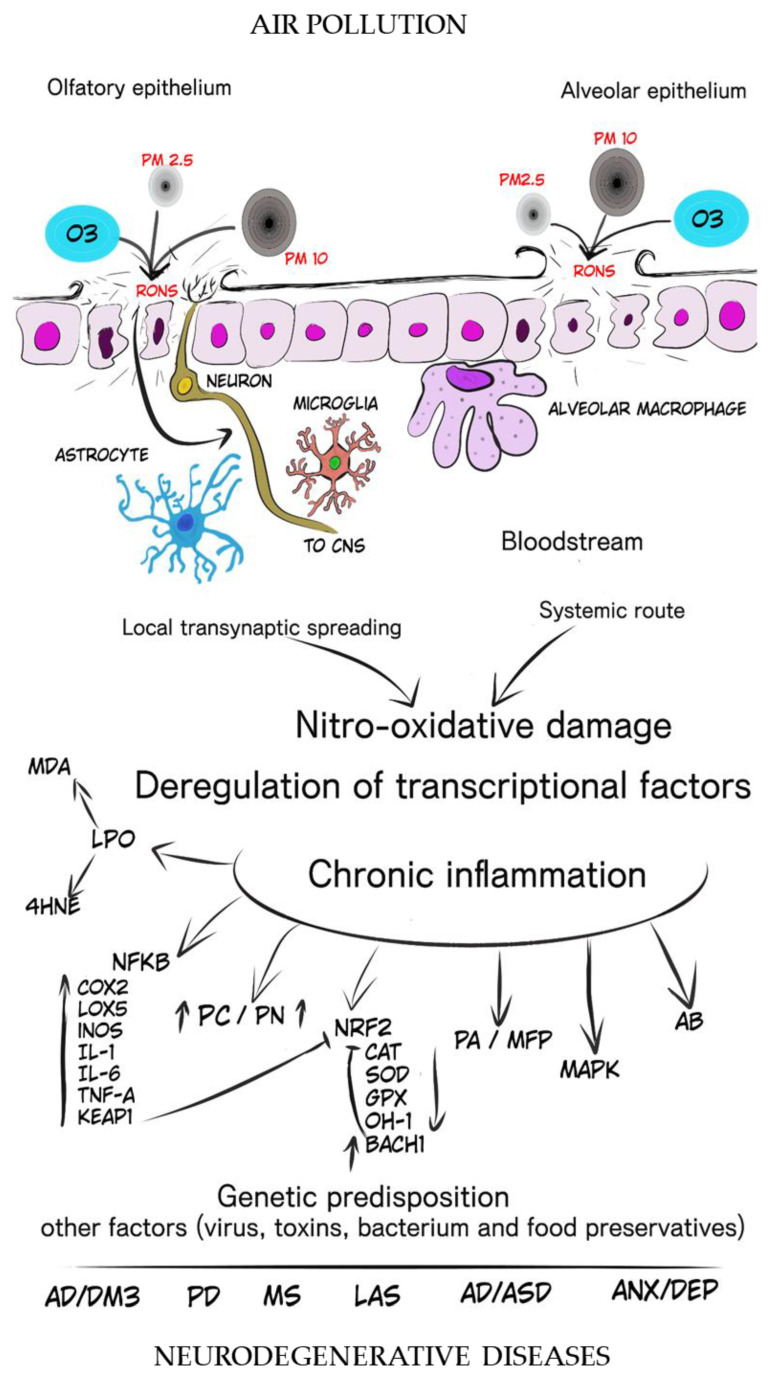
This image illustrates the participation of PMs and O_3_ with a landscape of the process that may lead to the initiation and progression of degenerative changes where nitrooxidative stress and chronic inflammation disrupt the regulation of homeostasis in the central nervous system. Particulate matter 2.5 and 10 µm (PM_2.5_ and PM_10_), ozone (O_3_), reactive oxygen and nitrogen species (RONS), lipid peroxidation (LPO), malondialdehyde (MDA), 4-hydroxinonenal (4-HNE), nuclear factor kappa B (NFκB), cyclo-oxygenase 2 (COX2), lipo-oxygenase 5 (LOX-5), inducible nitric oxide synthase (iNOS), interleukin 1 (IL-1), interleukin 6 (IL-6), tumor necrosis factor alpha (TNF-α), Kelch-like ECH-associated protein 1 (KEAP-1), protein carbonylation (PC), protein nitrosylation (PN), nuclear factor erythroid-related factor 2 (Nrf2), catalase (CAT), superoxide dismutase (SOD), gluthation peroxidase (GPx), heme-oxidase 1 (HO-1), BTB domain and CNC homolog 1 (BACH 1), protein accumulation (PA), misfolded protein (MFP), mitogen-activated protein kinase (MAPK), amyloid β (Aβ), Alzheimer’s disease (AD), diabetes mellitus type 3 (DM3), Parkinson’s disease (PD), lateral amyotrophic sclerosis (LAS), autism disorder/autism spectrum disorder (AD/ASD), anxiety/depression (ANX/DEP).

Summarizing the noxious effects of O_3_ we refer to Table 2 which illustrates the literature reviwed. Additionally, Figure 1 is schemetize that depicts the roles of PMs and O_3_ in the process that may lead to the initiation and progression of degenerative changes with emphasis in the nitrooxidative stress and chronic inflammation that disrupt the regulation of homeostasis in the central nervous system.

## 12. Prevention and Retardation of CNS Damage

The environmental policies established by the WHO to limit the health risks of atmospheric pollutants are applied with modifications of permissive limits in different countries. In general, policymakers redact laws and regulations in ambiguous language, and these are applied with lassitude, contributing to preserving bad air quality that leads to an increased prevalence of chronic neurodegenerative diseases. Strong economic pressure has retarded the use of advanced technologies to reduce the consumption of fossil fuels by internal combustion vehicles and industries [103]. Policymakers in health should consider recommendations for safe antioxidant consumption to prevent and/or revert the damage caused by air pollutants [99].

The scarce availability of pharmacological resources based on synthetic or natural molecules has acquired great scientific interest. Vitamin C supplementation exhibited a significant decrease in IL-6, TNF-α, and C-reactive protein levels, and it increased the glutathione peroxidase levels in humans exposed to PM_2.5_ (164.9 µg/m^3^) and PM_10_ (327 µg/m^3^) [104]. A review performed by Péter, et al. [105] analyzed the antioxidant and anti-inflammatory effects of vitamins C, D, and E, as well as omega-3 fatty acids against air pollution [105]. Vitamin E decreased the asthmatic inflammation caused by O_3_ through the reactivation of Nrf2 in mice [106]. Another study reported that vitamin E is capable of delaying aging in the brain and hepatic tissue through the reduction of oxidative stress in a murine premature aging model [107].

We have conducted a series of studies to analyze the preventive and therapeutic effect of curcumin (CUR) in a brain injury model caused by acute and chronic exposure to O_3_ (0.7 ppm, during 4 h for 15 or 60 days) in the hippocampi of Wistar rats [11,108,109]. First, we reported the antioxidant and anti-inflammatory effects of CUR (5.6 mg/kg, adsorbed in food), as it significantly decreased lipid peroxidation, protein carbonylation, IL-1, IL-6, and TNFα, and decreased NFκB activation exhibiting a better performance in the preventive approach [11]. In a further report using the same experimental approach, the neuroprotective effect of dietary CUR against the brain damage caused by O_3_ was documented. CUR decreased the astrocytosis, microgliosis, and neuronal apoptosis caused by O_3_ in the hippocampal regions CA1 and CA3 of rats [108]. Later, we informed the effect of dietary CUR on the activity level of antioxidant enzymes CAT, SOD, and GPx, and these effects are also related to the inhibition of lipid peroxidation and protein carbonylation [109]. Our latest report described the effect of CUR orally administrated in a preventive approach on the neurodegeneration and nitrooxidative damage caused by short-term exposure to O_3_ in a short-term scheme. The noxious effect of O_3_ (0.7 ppm) was evaluated at 1, 2, 4, and 8 h of exposure time with significant differences starting at 2 h and at 8 h the damage was more severe. The protective effect of CUR prevented the formation of polyamines, which are present in the cellular degeneration process, in hippocampal regions CA1 and CA3. These results were confirmed with the detection of amorphous disintegrative cellular debris by the de Olmos modified silver stain. Furthermore, the nitrooxidative damage was evaluated by lipid peroxidation, protein carbonylation and protein nitrosylation (3-nitrotyrosine), and CUR decreased it by about 50% [64].

Vastegani, et al. [110] reported the antioxidant effect of CUR at a dose of 50 mg/Kg of body weight in rats before the exposure to dusty PM (2000–8000 µg/m^2^, where PM_10_, PM_2.5_, and PM_1_ were present) in daily sessions of 60 min for 2 weeks. The oxidative damage and neuronal loss significantly decreased in the brain cortex and different areas of the hippocampus. The treatment with CUR also attenuated memory impairment, decreased anxiety and depression improving locomotor exploratory performance. Additionally, the treatment with CUR significantly decreased the BBB permeability [110].

Astaxanthin (marine red carotenoid) decreased neuroinflammation provoked by PM_2.5_ through regulation of Akt phosphorylation in a cultured BV-2 microglial cell line. Proinflammatory markers iNOS and HO-1, including the transition of microglial cells to M1 or disease-associated microglia markers were inhibited, thus astaxanthin kept microglial cells at a resting state [111].

Gallic acid (polyphenol from grapes, walnuts, and green tea), through its antioxidant and anti-inflammatory activities, decreased BBB permeability and MDA levels in ischemic rats exposed to PM [112].

## 13. Conclusions

As shown in this paper and other reviews, the close relationship between the damage caused by air pollutants and neurodegenerative markers is well recognized in AD, PD, and other diseases, and thus deserves intense investigation. Despite the harmfulness of air pollutants, PMs, and O_3_, and the ineffective regulations worldwide established by government agencies, the most reliable means to reduce brain damage caused by such pollutants is dietary supplementation with safe antioxidants from natural or synthetic origin. However, there is a reduced number of scientific reports that analyze this topic in experimental models or clinical assays. Particularly, clinical assays conducted to date are not conclusive in most cases, and many of them should be repeated with refined improvements in nutraceutical/pharmaceutical formulation technology, study design, volunteer selection, and analytical methods, among other specifications.

## Figures and Tables

**Table 1:** Summary of the effects of PMs in animal models and human populations.

Pollutant	Model	Concentration or Dose	Damage	Disease Markers Signaling Factors	Diseases	References
PM_2.5_ Beijing, China	ApoE-/- mice	Intranasal 4 mg/kg Single or repeated	Nitrooxidative stress	NO ↑, iNOS ↑, MMP9 ↑ TIMP-1 ↑, ICAM-1 ↑ TNF-α ↑, IL-6 ↑ MDA ↑, SOD ↓	Undefined	Long et al., 2020 [20]
PM_2.5_ Diesel NIST standard 2975, Merck	Male BALB/c mice	Intranasal 100 µg/40 µL per animal. (OVA 400 µg/20 µL) per animal	Oxidative stress Inflammation	MDA ↑,SOD ↓, HO1 ↓ NFκB ↑, Nrf2 ↓, IL-4 ↑ IL-5 ↑, IL-13 ↑, IL-17 ↑ IL-17 ↑, TGF-β1 ↓	Allergic Rhinitis	Piao et al., 2021 [21]
PM_2.5_ Tangshan City, China	Sprague–Dawley male rats	Intratracheal 20 mg/kg/7 days (3, 6 and 12 months)	Behavioral tests Apoptosis Oxidative stress	Spatial learning and memory ability ↓ Inquiring ability ↓ Sensory function ↓ SOD and GSH-Px ↓ MDA ↑, myelin sheath integrity ↓, apoptosis↑ mitochondrial damage ↑	Aging AD PD MS	Zhang et al., 2018 [22]
Ambient dusty PM	Male Wistar rats	Ambient PM inhalation 200–500 μg/m^3^ 500–2000 μg/m^3^ 2000–8000 μg/m^3^ 30 min/twice daily/4 weeks	BBB Electrophysiology Edema Histology Behavior Oxidative stress Inflammation	BBB permeability ↑, Edema ↑, spatial learning, and memory ↓ LTP ↓, MDA ↑, GPx ↓ TNF-α ↑, IL-10 ↓	AD, PD ADHD COPD ASD, ANX Stroke N-AD	Hajipour et al., 2019 [23]
MCMA ambient PM_10_ PM_2.5_	Mongrel dogs (15 M/25 F)	PM_10_ 78 µg/m^3^ PM_2.5_ 21.6 µg/m^3^	Apoptosis Nitrooxidative stress Inflammation	TUNEL glial cells ↑, Astrocytosis(GFAP+) ↑ COX 2 ↑, iNOS ↑, NFκB ↑, ApoE ↑, Aβ ↑ Reactive microgliosis ↑	AD PD	Calderon-Garciduenas et al., 2003 [31]
MCMA FPM < 2.5 PM_2.5_	School children (11.69 yo)	PM_2.5_: 22.3 to 16.8 μg/m^3^ O_3_: 0.165 to 0.129 ppm 8 h exposure	Metabolism	ET-1, leptin, glucose ↑ Ghrelin, GLP-1 ↓, glucagon ↓, insulin ↑, APOE 4 higher glucose vs. APOE 3	AD	Calderon-Garciduenas et al., 2016 [4]
PM_2.5_ > 10 µg/m^3^	Elderly over 65 y	Ambient exposure	Odds ratios (OR) Hospital admission	AD mortality ↑, non-AD ↑, PD ↑ OR for AD > non-AD or PD	AD Non-AD PD	Rhew et al., 2021 [35]
PM_2.5_ UFPM 8.1 μg/m^3^	Mean age 64.3 y ± 13.5 6655 patients 45.3% F 54.7% M	8.1 μg/m^3^ OM 35% SO_4_^−^ 31% HNO_3_ 0.9% BC 0.6 Soil 0.3% SS 0.2%	Hospital admissions	ALS 41.0% (motor complication, respiratory failure) RD 16.0% Non-ALS (infections, heart attack)	ALS aggravation outcome	Nunez et al., 2022 [36]
PM_10_	Female: Male Ratio 2.75 Age 30.7 ± 11.7 y	Ambient peak exposure Over 50 µg/m^3^	536 patients admitted for MS relapses Annualized relapse rate 0.57/y 0.47 men; 0.60 women	Positive association PM_10_ exposure and risk of MS relapse	Increased natural log of average 1 to 3 days before relapse OR = 1.40 in cold season	Roux et al., 2017 [38]
PM_10_, O_3_ CO, NO_2_ SO_2_	49,073 children under 3 y	Ambient exposure for 10 y	Children diagnosed with ASD	Positive association with O_3_ in 10 ppb increase; CO 100 ppb, NO_2_ 10 ppb. No association with PM_10_	342 children newly diagnosed with ASD 83.2% male	Jung et al., 2013 [39]
PM_10_, PM_2.5_	Children born between 1990 and 2002	Maternal ambient exposure during pregnancy	Diagnosed with ASD	Association between PM_2.5_ exposure during third trimester and ASD 1.42; exposure during first and second trimesters and ASD was 1.06	245 Diagnosed with ASD	Raz et al., 2015 [40]
PM_2.5_ NO_2_	Children born between February 2004 and 2008	Maternal ambient exposure 1119 women	Cognition Verbal Numeric Motor	Memory deficit in boys associated with PM_2.5_ and NO_2_. Global cognition and verbal expression associated with NO_2_.	Sex-dependent effects at 4–6 years of age	Lertxundi et al., 2019 [43]
UFPM Nano BC 14 nm	BALB/c mice	Intranasal instillation olfactory bulbs 250 μg 100 µL	Neurotransmitter release Inflammatory markers	Glutamate and Glycine ↑ IL-1β and TNF-α ↑	AD PD	Tin Tin Win et al., 2008 [53]
PM_2.5_ O_3_ Southwest Mexico City	Children 96.3 ± 8.5 months of age 34 from highly polluted 17 from low pollution Autopsy material from 5 subjects	PM_2.5_ 24.6 µg/m^3^ vs. 15 µg/m^3^ of USA National Ambient Air Quality Standard Maximum average 90 µg/m^3^ during the mid-morning	Brainstem auditory evoked potentials (BAEPs) Inflammatory markers AD markers Inflammatory markers	Delayed conduction time of brainstem neural transmission Vestibular impairment IL-1β, TNF-α, TGF-β_1_, MDC, MCP-1 ↑; IL-8 ↓, Medial superior olive neurons: α synuclein +, reactive microgliosis, βA_1–42_ +	Diverse Neuropatho logies	Calderón-Garcidueñas et al., 2011 [54]
Ambient PM_2.5_ UFPM_0.1_	C57BL/6 male mice	PM_2.5_ ≈ 70 µg/m^3^ UFPM_0.1_ ≈ 10,000–20,000/m^3^	PM detection Neuroinflammation AD hallmarks	PM particles in brain tissue + BBB disruption Enlargement of perivascular space Inflammatory cells attached to brain vascular endothelium Aβ plaque formation Reactive microgliosis	AD	Hameed et al., 2020 [55]
PM_2.5_ Taiyuan, China	C57BL/6 male mice	Oropharyngeal aspiration 1 and 5 mg/kg Every other day for 4 weeks Intracerebral injection with BACE1, shRNA or LV-miR-574-5p	Neuroinflammation Synaptic function Spatial learning memory	BACE1 overexpressed Aβ generated NFκB activation by nitrooxidative stress	AD	Ku et al., 2017 [56]
PM O_3_, SO_2_ NO_2_, CO Pb	139 children Mean age 11.91 ± 4.2 years	Ambient exposure MCMA	Systemic inflammation Neuroinflammation	MIF, IL-6, IL-1ra, IL-2, PrP^C^ ↑	AD PD	Calderón-Garcidueñas, et al., 2013 [60]

**Table 2:** Summary of literature reviewed.

Pollutant	Model	Concentration or Dose	Damage	Disease Markers Signaling Factors	Disease	References
O_3_ O_2_ passed through an ozone generator	21 days old male Wistar rats	Inhaled 0.7 ppm 1, 2, 4, and 8 h exposure	Nitrooxidative stress Neurodegeneration	Protein nitrosylation ↑ Protein carbonylation ↑ Polyamine accumulation and amorphous anionic debris in hippocampal neurons ↑	AD Other Neurodegenerative Diseases	Mendoza-Magana et al., 2021 [64]
O_3_ O_2_ passed through an ozone generator	21 days old male Wistar rats	Inhaled 0.7 ppm Daily exposure for 4 h, 15 and 60 days	Oxidative stress	MDA and 4-HNE ↑ NFκB activation ↑, IL-1β and TNF-α ↑ Protein carbonylation ↑	Neurodegenerative Diseases	Nery-Flores et al., 2018 [11]
O_3_ Filtered purified air passed through an ozone generator	Male Wistar rats 250–300 g/bw	Inhaled 0.25 ppm Daily exposure for 4 h, 15, 30, 60, and 90 days	Peptide conformational changes Aβ_1–42_ immunodetection	Time-dependent effect α-helix secondary structure ↓, β-sheet secondary structure ↑, Aβ_1–42_ neuronal deposition ↑	AD	Rivas-Arancibia et al., 2017 [76]
O_3_ Filtered purified air passed through an ozone generator	Male Wistar rats 250–300 g	Inhaled 0.25 ppm Daily exposure for 4 h, 7, 15, 30, 60, and 90 days	Endoplasmic reticulum stress Apoptosis	ATF6 ↑, GRP78 ↑, caspase 12 ↑ TUNEL + cells ↑	AD	Rodrigues-Martinez et al., 2016 [77]
O_3_ Filtered purified air passed through an ozone generator	Male Wistar rats 250–300 g	Inhaled 0.25 ppm Daily exposure for 4 h, 7, 15, 30, 60, and 90 days	Protein oxidation Microgliosis Astrocytosis In substantia nigra	Protein carbonylation ↑ Cit C ↑, GFAP (30 and 60 days) ↑, Iba-1 (60 days) ↑, COX-2 ↑, Nuclear NFκB ↑	PD	Rivas-Arancibia et al., 2015 [79]
O_3_ Filtered purified air passed through an ozone generator	Male Wistar rats 250–300 g	Inhaled 0.25 ppm Daily exposure for 4 h, 7, 15, 30, 60, and 90 days	Aβ_1–42_ and _1–40_ expression Aβ mitochondrial accumulation	Aβ_1–42_ accumulated in mitochondrial fraction and inside the organelle. Pres2 ↑, ADAM 10 ↓.	AD	Hernández-Zimbrón et al., 2015 [86]
O_3_ Filtered purified air passed through an ozone generator	Male Wistar rats 250–300 g	Inhaled 0.25 ppm Daily exposure for 4 h, 15, 30, 60, and 90 days	Density and morphology of dendritic spines in CA1 hippocampal region. Learning and memory	Density of dendritic spines ↓ Thin and mushroom spine ratio ↓ Stubby spine ratio ↑ Object–place recognition ↓	AD	Bello-Medina et al., 2019 [90]
O_3_ Filtered purified air passed through an ozone generator	Male Wistar rats 250–300 g	Inhaled 0.25 ppm Daily exposure for 4 h, 15, 30, and 60 days	Dopaminergic cell count Dopamine oxidation Oxidative stress	Dopaminergic cell count ↓ Dopamine quinones ↑ LPO ↑, p53 + cells ↑	PD	Santiago-López et al., 2010 [91]
Ambient O_3_ and PM_2.5_	5116 subjects normal cognition possible/probable AD Period 2005–2008	Ground level inhalation PM_2.5_ 9.7 ± 1.9 µg/m^3^ Under NAAQS O_3_ 36.7–40 ppb	Cognitive decline	PM_2.5_ was not associated with the rate of cognitive decline Increased O_3_ concentration correlated with increased rate of cognitive decline	AD	Cleary et al., 2018 [93]
O_3_ Generated Passing filtered air through UV light	Wistar rats 400–450 g	Inhaled 0.8 ppm For 16 h	Insulin resistance Signaling pathways Oxidative stress	Fasting blood glucose and insulin concentration ↑ HOMA-IR ↑, insulin-induced protein kinase B (PKB)/Akt phosphorylation ↓ in muscle, but not in hepatic or adipose tissue Activation of JNK ↑ HHE, HNE, and MDA ↑ in BALF and muscle Protein carbonylation ↑ GSH-to-GSSG ratio ↓	DMT2	Vella et al., 2015 [97]
O_3_ Generated Passing filtered air through UV light	Male Sprague–Dawley rats 280–320 g	Inhaled 0.5 ppm/3 h	Neuroprotection Nucelous tractus solitarius and ventrolateral medulla	VEGF expression, IL-6, TNF-α, and GFAP ↑	Undefined	Araneda et al., 2008 [102]

## Glossary

Aβ	Amyloid beta
AD	Alzheimer’s disease
ADHD	Attention deficit hyperactivity disorder
AGES	Advanced glycosylation end products
ALS	Amyotrophic lateral sclerosis
AQGL	Air quality guidelines level
ARE	Antioxidant response elements
ASD	Autism spectrum disorder
ATP	Adenosine triphosphate
BACE1	Beta site amyloid precursor protein
BBB	Brain–blood barrier
BDNF	Brain-derived nerve growth factor
CAT	Catalase
CNS	Central nervous system
COX-2	Cyclooxygenase 2
CUR	Curcumin
EADS	Endogenous antioxidants enzyme system
ER	Endoplasmic reticulum
F2-isoPs	F2 isoprostane
GFAP	Glial fibrillary acidic protein
GPx	Glutathione peroxidase
GR	Glutathione reductase
HOMA-IR	Homeostasis Model Assessment–Insulin resistance
HO-1	Heme-oxygenase 1
H_2_O_2_	Hydrogen peroxide
IDE	Insulin-degrading enzyme
Iκβ	Inhibitor of NFκβ
Iκκ	Inhibitor kappa β kinase
iNOS	Inducible nitric oxide synthase
IL-1	Interleukin 1
IL-6	Interleukin 6
IL-10	Interleukin 10
IR	Insulin resistance
IRs	Insulin receptors
JNK	Janus kinase
KEAP1	Kelch-like ECH-associated protein 1
LPS	Lipopolysaccharide
LTP	Long-term potentiation
LOX	Lypooxigenase
MDA	Malondialdehyde
MMC	Mexico Metropolitan City
mtDNA	Mitochondrial deoxyribonucleic nucleic acid
MS	Multiple sclerosis
N-AD	non-Alzheimer’s dementia
NFκB	Nuclear factor kappa B
NGF	Nerve growth factor
NH_3_	Ammonia
NOS	Nitrooxidative stress
NO_2_	Nitrogen dioxide
NO	Nitric oxide
NOx	Nitrogen oxides
Nrf2	Nuclear factor erythroid-related factor 2
NS	Nitrosative stress
ONOO^−^	Peroxynitrite
OS	Oxidative stress
O^2−^	Superoxide anion
O_3_	Ozone
PD	Parkinson’s disease
PM	Particulate matter
PM_2.5_	Particulate matter 2.5 µm
PM_10_	Particulate matter 10 µm
ppb	Parts per billion
ppm	Parts per million
REDOX	Reduction/oxidative
RONS	Reactive oxygen and nitrogen species
SOD	Superoxide dismutase
SO_2_	Sulfur dioxide
TNFα	Tumor necrosis factor alpha
TRAF	TNF receptor-associated factor
UFPM	Ultrafine particulate matter
UV	Ultraviolet
VEGF	Vascular endothelial growth factor
VOCs	Volatile organic compounds
WHO	World Health Organization
3-NT	3-nitrotyrosine
4HNE	4-hydroxinonenal
